# Single‐Cell Mitochondrial DNA Analysis of Recombinant Chinese Hamster Ovary Cells Reveals Widespread Heteroplasmy

**DOI:** 10.1002/biot.70079

**Published:** 2025-09-01

**Authors:** Alan Foley, Nga Lao, Ciara McGuirk, Colin Clarke, Niall Barron

**Affiliations:** ^1^ Cell Engineering Group NIBRT Dublin Ireland; ^2^ School of Chemical and Bioprocess Engineering University College Dublin Dublin Ireland; ^3^ Bioinformatics Group NIBRT Dublin Ireland

**Keywords:** CHO, heteroplasmy, mtDNA, scmtDNAseq, single cell

## Abstract

Recent bulk analysis of Chinese hamster ovary (CHO) cell mitochondrial DNA revealed widespread heteroplasmy across cell lines and even within clones of the same parental host. To address this, we applied our previously developed single‐cell mtDNA sequencing (scmtDNAseq) method to 84 single CHO cells. We identified widespread intercellular heteroplasmy across the CHO cell population and predicted possible phenotypic impacts. 3/11 (27%) of the most variable mutations were only identified by scmtDNAseq, indicating greater resolution when compared to bulk cell analysis. Single‐cell RNAseq (scRNAseq) was also performed at the same time point and, compared to scmtDNAseq, significant differences in intercellular heteroplasmy were observed. Using an inducible mAb expression system demonstrated that short‐term additional biosynthetic burden of exogenous protein production had little impact on intercellular heteroplasmy. We additionally monitored bulk heteroplasmy over 38 days, reflecting the typical timespan from vial thaw to production vessel in a Biopharmaceutical upstream cell culture process. We observed minimal change in heteroplasmy, finding no evidence that a mAb‐producing CHO cell line develops impactful changes in heteroplasmy over that timeframe. This would suggest that for our cell line, the heteroplasmy profile established on Day 1 should be maintained throughout a full fed‐batch bioprocess run.

## Graphical Abstract and Lay Summary

Single‐cell mtDNA sequencing of Chinese hamster ovary (CHO) cells revealed widespread intercellular heteroplasmy. Compared to bulk samples, single cells exhibited unique mutations, highlighting greater resolution. The “switching on” of mAb production had minimal impact on heteroplasmy. Bulk samples over a 38 fed‐batch process showed stable heteroplasmy, suggesting Day 1 profiles persist. Single‐cell RNA sequencing (scRNAseq) corroborated some mutations observed in DNA sequencing and suggested possible RNA editing events.

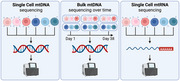

**Abbreviations**
ATP: adenosine triphosphate
CHO: Chinese hamster ovary
CHO‐PVZ: palivizumab‐producing CHOBRI/55E1 cell line
DAPI: 4′,6‐diamidino‐2‐phenylindole
FACS: fluorescence‐activated cell sorting
IgG: Immunoglobulin G
LRPCR: long‐range PCR
mAb: monoclonal antibody
mtDNA: mitochondrial DNA
NRC: National Research Council Canada
Numts: nuclear mitochondrial DNA
OXPHOS: oxidative phosphorylation
PBMCs: peripheral blood mononuclear cells
qP: specific productivity
scATACseq: single‐cell assay for transposase‐accessible chromatin using sequencing
scmtDNAseq: single cell mtDNA sequencing
scRNAseq: single‐cell RNA sequencing
TALENs: transcription activator‐like effector nuclease
TCA cycle: tricarboxylic acid cycle
WGS: whole genome sequencing

## 1 Introduction

In contrast to the nuclear genome, mitochondria contain multiple copies of their genomic DNA (mtDNA). Mutations in mtDNA can lead to a state of “heteroplasmy” where a proportion of mutated mtDNA copies co‐exist with wild‐type copies. The phenotypic effect of heteroplasmy is well demonstrated in primary mitochondrial disorders such as certain forms of Leigh syndrome, a neurodegenerative disorder affecting children [1]. As the proportion of mutant copies of mtDNA increases, the severity of symptoms increases, reflecting further impairment of mitochondrial function [2]. On the other hand, below a phenotypic allele frequency threshold, no phenotypic effect is observed.

Phenotypic heterogeneity is a well‐recognized feature of therapeutic protein‐producing Chinese hamster ovary (CHO) cell lines necessitating clone screening and selection during the generation of transgenic CHO cell lines [3]. Clones derived from the same pool of transfected cells can have highly variable phenotypes such as recombinant protein yield, growth rate, or culture viability. Mitochondrial function likely contributes to this phenomenon; presenting an opportunity to screen for and select those with the most desirable features. Indeed, selection of cells with higher mitochondrial membrane potential, an indicator of mitochondrial activity, led to increased product titer [4]. However, concomitant with this heterogeneity can be a tendency towards phenotypic instability, which can be problematic once a producer clone is selected [5]. Biopharma companies are therefore compelled to screen any chosen clone to demonstrate that their producer clone is stable and hence makes a consistent biotherapeutic product. It is possible that mitochondrial heteroplasmy could also contribute to this via perturbed metabolic pathways, e.g., the citric acid (TCA) cycle or oxidative phosphorylation (OXPHOS).

This heterogeneity may involve the Warburg effect, which describes how exponential cell growth can lead to increased aerobic glycolysis—as observed in cancer cells [6]. This less efficient energy process bypasses the mitochondrial‐based TCA cycle and OXPHOS. The resulting waste metabolite lactate can lead to a plateau in further cell growth and productivity [7]. This finding led researchers to engineer CHO cell lines with more favorable bioprocessing qualities. For example, when lactate production was reduced by 40% in a CHO cell fed‐batch culture, the antibody titer doubled [8]. Furthermore, metabolic variability in replicate bioreactors has previously been attributed to changes in lactate production rates—a factor mitigated by mitochondrial TCA cycle intermediate supplementation [9].

Previous bulk CHO cell mtDNA analysis revealed significant heteroplasmy between CHO cell lines and even between clones from the same parental host [10]. However, this type of bulk analysis describes an “averaged” cell, which may not represent any individual cell within the population. We previously reported a method to analyze mtDNA in individual CHO cells via single‐cell mtDNA sequencing (scmtDNAseq) [11]. Thus, we sought to expand our investigation by applying this approach to a larger panel of cells to better decipher the extent of mitochondrial heteroplasmy in this commercially important cell line.

**FIGURE 1:**
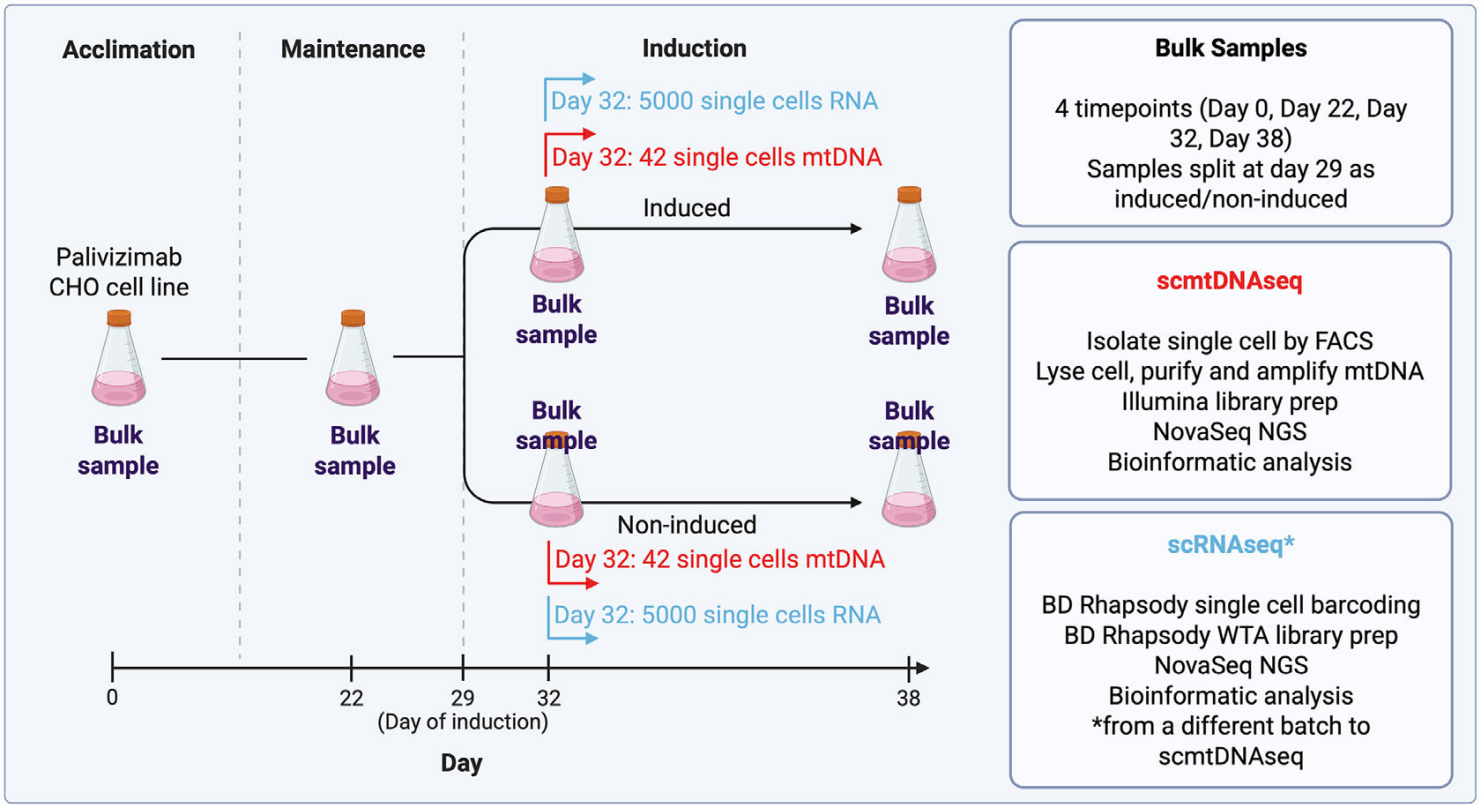
Experimental overview. An inducible, clonally‐derived CHO cell line producing the monoclonal antibody Palivizumab (CHO‐PVZ) was cultured for 38 days in a fed‐batch culture. Bulk samples were taken intermittently to investigate the impact of time in culture on mtDNA heteroplasmy. After induction, samples (both bulk and single cell) were split between induced and noninduced. Single cells were captured at day 32 from flasks, 3 days after inducing mAb production or an uninduced control for both scmtDNAseq and scRNAseq. scRNAseq samples came from the same process but different batches.

In this study, bulk samples were taken along a time scale typical of a CHO cell bioreactor campaign (Figure 1). This ensured we captured any changes in heteroplasmy that could conceivably impact a population of CHO cells over a “real world” manufacturing timeframe, including seeding the culture, through scale‐up to production. In parallel to bulk analysis, the full mtDNA genomes from 84 single cells of a recombinant protein‐producing CHO cell line were interrogated by scmtDNAseq. Key to the experimental design was the use of an inducible CHO cell line, which enabled investigation of the impact of “switching on” mAb production, in the same genetic background [12] rather than a comparison with independent, nonproducing parental cells. Finally, as an orthogonal approach, we performed single‐cell RNA sequencing (scRNAseq) to compare it to scmtDNAseq in its ability to identify genomic mutations and concomitantly identify nongenomic mutations such as RNA editing events.

## 2 Materials and Methods

### 2.1 Cell Culture

The CHOBRI/55E1 cell line (CHO‐PVZ) stably transfected with an inducible palivizumab (PVZ)‐expressing plasmid was a kind gift from the Biotechnology Research Institute of the National Research Council Canada (NRC). A vial was thawed from liquid nitrogen on Day 0 and added directly to 10 mL of 37°C PowerCHO2 (LONZA 12–771Q). After brief centrifugation at 200 × *g*, the supernatant was discarded, and the pellet was resuspended into 30 mL of fresh PowerCHO2 + 50 µM MSX (Sigma M5379) in a 125 mL vented culture flask (FisherScientific 102664332). Cells were maintained at 37°C, 5% CO_2_ in a Kuhner orbital shaker platform at 125 rpm. Cells were passaged 3 times per week, seeding at 0.3 * 10^6^ cells/mL for a 2‐day passage interval or 0.15 * 10^6^ over the 3‐day weekend.

### 2.2 Induction of Recombinant Protein Expression

On Day 26 (3 days before induction), 6 flasks of CHO‐PVZ were seeded at 0.3 * 10^6^ cells/mL in BalanCD CHO (Irvine Scientific 91128) + MSX 50 µM. On Day 29 (the day of induction), the 6 flasks were mixed and distributed evenly among 6 new flasks at 2.5*10^6^ cells/mL. Triplicate flasks were induced and the other three were maintained as noninduced controls. All flasks were placed in the same conditions as described above, but with the temperature shifted to 32°C. To induce antibody expression, cumate was added to a final concentration of 2 µg/mL. To all flasks, Feed F12.7 solution (Irvine Scientific 98944) was added at 5%, 5%, 10%, 15%, and 10%(v/v) on days 29, 32, 34, 36, and 38, respectively. Anticlumping agent (FisherSci 15234475) was added at each timepoint (300 µL). Glucose was monitored using a Verio Reflect Glucose Monitor and cell density was measured by trypan blue exclusion. Where cell clumping was observed, 300 µL of a cell culture sample was added to 300 µL of ACCUMAX (Innovative Cell Technologies AM105), incubated with agitation at 37°C for 30 min and then cells were counted. At each time point, 230 µL of supernatant was stored at −80°C for subsequent Immunoglobulin G (IgG) quantification by Octet R2 using Protein A Biosensors (forteBIO 18–5010).

### 2.3 ScmtDNAseq

Three days after induction (Day 32), cells were stained at 4°C with an anti‐IgG AB‐FITC conjugate (Sigma Aldrich SAB3701254‐2MG), which binds to the palivizumab in stasis in the cell membrane, in a process known as cold capture (Figure S1A) [13]. Maintaining samples on ice, cells were resuspended in DPBS + DAPI 0.1 µg/mL. Single cells that were AB‐FITC (+) and DAPI (‐) were sorted in a BD FACS Melody using “single cell mode” into 5 µL of TCL (QIAGEN) lysis buffer in a U‐bottom 96‐well plate. Due to leaky palivizumab expression in noninduced cells, both induced and noninduced could be sorted using an AB‐FITC tag. Bulk samples (10,000 cells) were also sorted using the “purity” setting on the FACS Melody. From here, we followed our previously published method [11], amplifying 2 fragments in long‐range PCR (Figure S2) except without Equimolar combination. Library prep was performed using Illumina DNA Prep (Illumina 20018705) and IDT for Illumina DNA/RNA UD Indexes Set A (Illumina 20027213) for a total of 94 samples: 88 single cells (44 from induced and 44 from noninduced), and 6 bulk samples. Samples were sequenced on a NovaSeq (Illumina) for 150 nt paired‐end (PE150) reads generating a total of 87Gb of raw data.

Due to the large number of samples being analyzed, we combined the separate fragments (X and Y) (Figure S2) after the PCR step in equal volumes, i.e., not necessarily in equimolar quantities. We reasoned that this may introduce some bias of coverage into the data since the PCR may have amplified each fragment differently. To investigate this, we took a segment of each fragment's (X and Y) coverage values. For the X fragment, we used positions 2000–7000, and for the Y fragment positions 10000–15000. This minimized the effect of overlapping primer regions on the analysis. We then averaged the per‐base depths for each fragment and removed any single cells which contained less than 1000 average per‐base depth in either fragment (Figure S1D). We generated a proportional bar chart that plotted the proportion of depth attributed to each fragment in each cell (Figure S3A). Most single cells had similar read depth between fragments. However, some single cells displayed considerably different fragment depth, for example, N41 (noninduced single cell 41) had over 80% of reads assigned to the Y fragment. The average absolute differential was 1370 reads and the standard deviation 1721. In total, 4 single cells were removed due to low coverage in either the X or Y fragment (2 induced, 2 noninduced), resulting in 84 total single cells being further analyzed. Bioinformatics was performed as previously described [11]. Further analysis was performed using dplyr and ggplot for visualization.

### 2.4 scRNAseq

Importantly, while the cell culture methodology was performed in the same way, the scRNAseq and scmtDNAseq data were generated from different cell culture runs; thus, the single cells did not come from the same vessel. Single cells were captured into nanowells and lysed as part of the WTA BD Rhapsody protocol (23‐21751‐00). Unlike the scmtDNAseq method, AB‐FITC was not used to select cells. The mRNA Whole Transcriptome Analysis (WTA) was followed (23‐21751‐00). Briefly, WTA libraries were separated into individual libraries, giving 2 final libraries: (1) PVZ Induced WTA, and (2) PVZ Noninduced WTA Libraries were quantified and pooled to be sequenced on an Illumina NovaSeq targeting a yield of 30,000 reads/cell.

STAR 2.5.2b was used to generate a reference genome file from the CHO CriGri‐PICRH‐1.0 reference genome [14] with added CHO mitochondrial reference KX576660.1 [14]. In addition, mitochondrial genes were added to the CriGri‐PICRH‐1.0 gtf file. These reference genome files, along with FASTQ files from the NovaSeq, were run on the Sevenbridges BD Rhapsody WTA analysis Pipeline revision 7 (v1.10.1); reads were trimmed and tagged with unique barcoded sequences for single cell identification.

Output BAM files from Sevenbridges were run on Sevenbridges BamTools Split (Revision 11) to subset only mtDNA reads. The “Seurat—Guided Clustering Tutorial” [15] was used to filter for only live and high‐quality cells, identified as mitochondrial reads < 17% and 2100 < nFeatureRNA < 4000, respectively (Figure S1E).

mtDNA BAM files contained extensive polyA contamination and base misalignment (Figure S5A). PolyA sequences with 5 or more consecutive As were removed using ClipReads (gatk‐4.2.6.1). Mapping realignment by STAR (2.7.10a) achieved much improved mapping. Cells were filtered out if they had lower than 50 average per‐base read depth. Only mutations with above 49 read depth were retained. Since mapping software assumes a linear reference genome, mutation calling was repeated with a shifted mtDNA reference genome to improve coverage over the D‐loop area. Unshifted sequence mutation calls were concatenated with shifted to provide full coverage. We used ggplot2 in R to generate figures and Student's *t*‐test to test for statistical differences between groups.

## 3 Results

### 3.1 Culture Duration Has a Negligible Impact on Mitochondrial Heteroplasmy in CHO Cells

**FIGURE 2:**
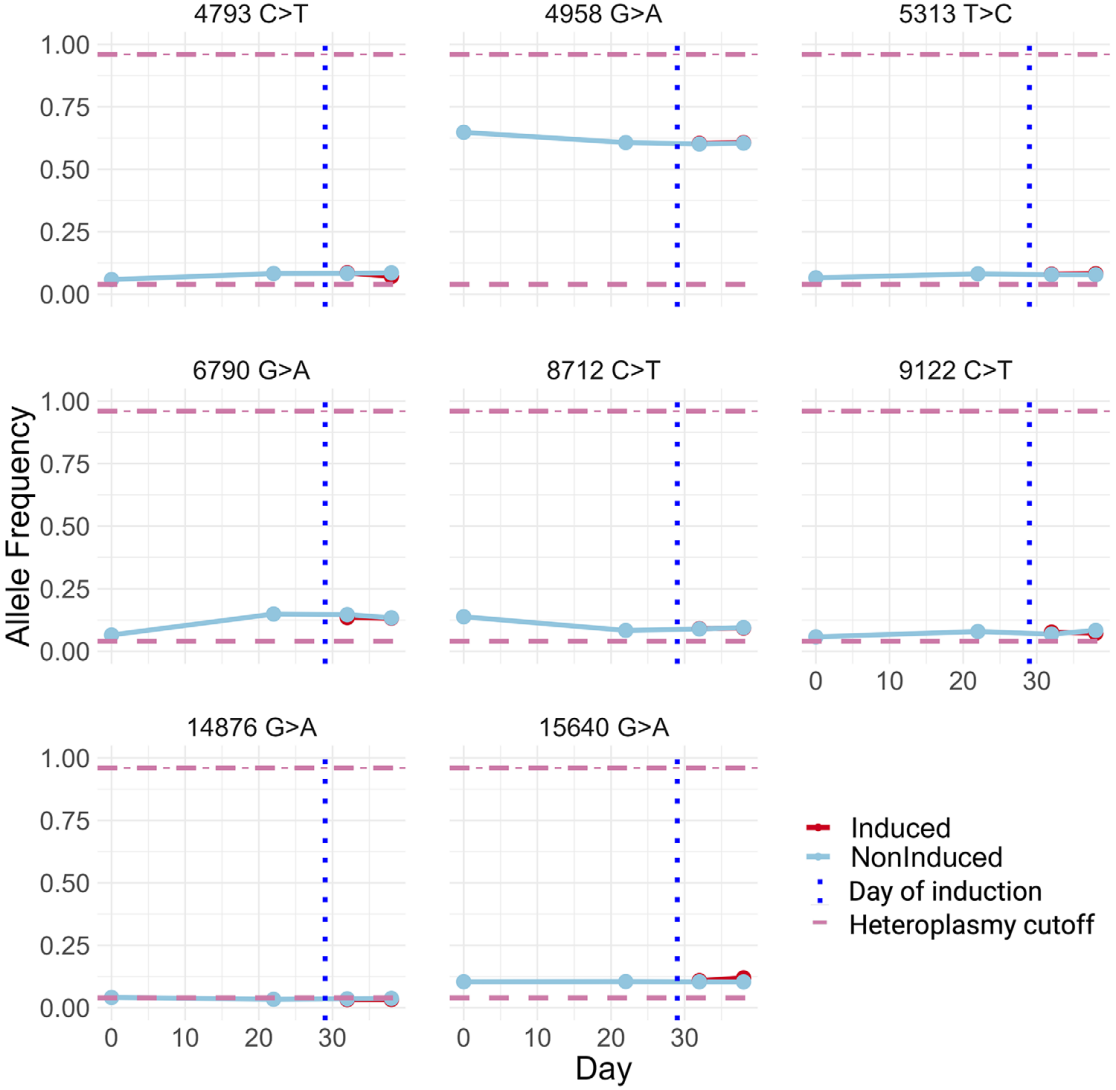
The bulk allele frequencies of 8 heteroplasmic mutations over a 38‐day clonally derived CHO cell culture.

To investigate whether CHO cell heteroplasmy changed over the duration of a typical bioreactor run, bulk samples of 10,000 cells were sequenced periodically along a 38‐day cell culture (Figure 1). Eight mutations were identified at all time points (Table S1) (Figure 2) within a 0.04‐0.96 heteroplasmy cutoff as previously used in the literature [11, 16, 17, 18]. A modest change in allele frequency was observed over time in the mutation 6790 G>A between 0.06 and 0.15. 4958 G>A fluctuated between 0.6 and 0.65, which might be considered to be approaching a phenotype‐impacting threshold [19]. For all other mutations, no notable changes in allele frequency were observed.

### 3.2 scmtDNAseq Identifies Differences in Heteroplasmy Between Individual CHO Cells

As a natural extension to bulk analysis, single CHO cells were analyzed 3 days post‐induction of mAb production to investigate intercellular heteroplasmy at a very high resolution. The average of single‐cell allele frequencies approximated the allele frequencies in the equivalent bulk sample (Figure S5B). This was more concordant than we previously observed [11], perhaps due to the greater number of single cells analyzed in this study. Interestingly, 6 of the 7 (86%) mutations displayed a slightly lower allele frequency in the single‐cell average when compared to the bulk. We reasoned that any mutations below a 0.04 frequency in a single cell would be counted as a “0” value, whereas in bulk samples, any particular mutant allele frequency would be an average across the population. Indeed, the only mutation with the same allele frequency in bulk and the single cell average was 4958 G>A, which occurs at a much higher allele frequency than most others at around 0.6; and would therefore be less likely to be affected in this way than mutations with a frequency around the 0.04 cutoff.

### 3.3 Detected mtDNA Mutations Are Predicted to Have Phenotypic Effects on CHO Cells

While heteroplasmic mutations in general represent a degree of intercellular heterogeneity, the phenotypic impact is influenced by 3 main factors: (1) mutations being in coding or noncoding regions, (2) mutations being above or below a phenotypic threshold, and (3) impact of mutations on amino acid sequence. Here, a more limited single cell predicted “most impactful” mutation list was chosen based on 2 criteria: (1) the mutation is present in at least 1 cell above 30% allele frequency—assumed to be a modest phenotypic threshold and (2) snpEff predicts a high or moderate impact; equating to stop‐gained, frameshift or missense mutations (Table S1). While we acknowledge that mutations not meeting these criteria could also have phenotypic effects, we focused on those “most likely to cause phenotypic effects.”

**FIGURE 3:**
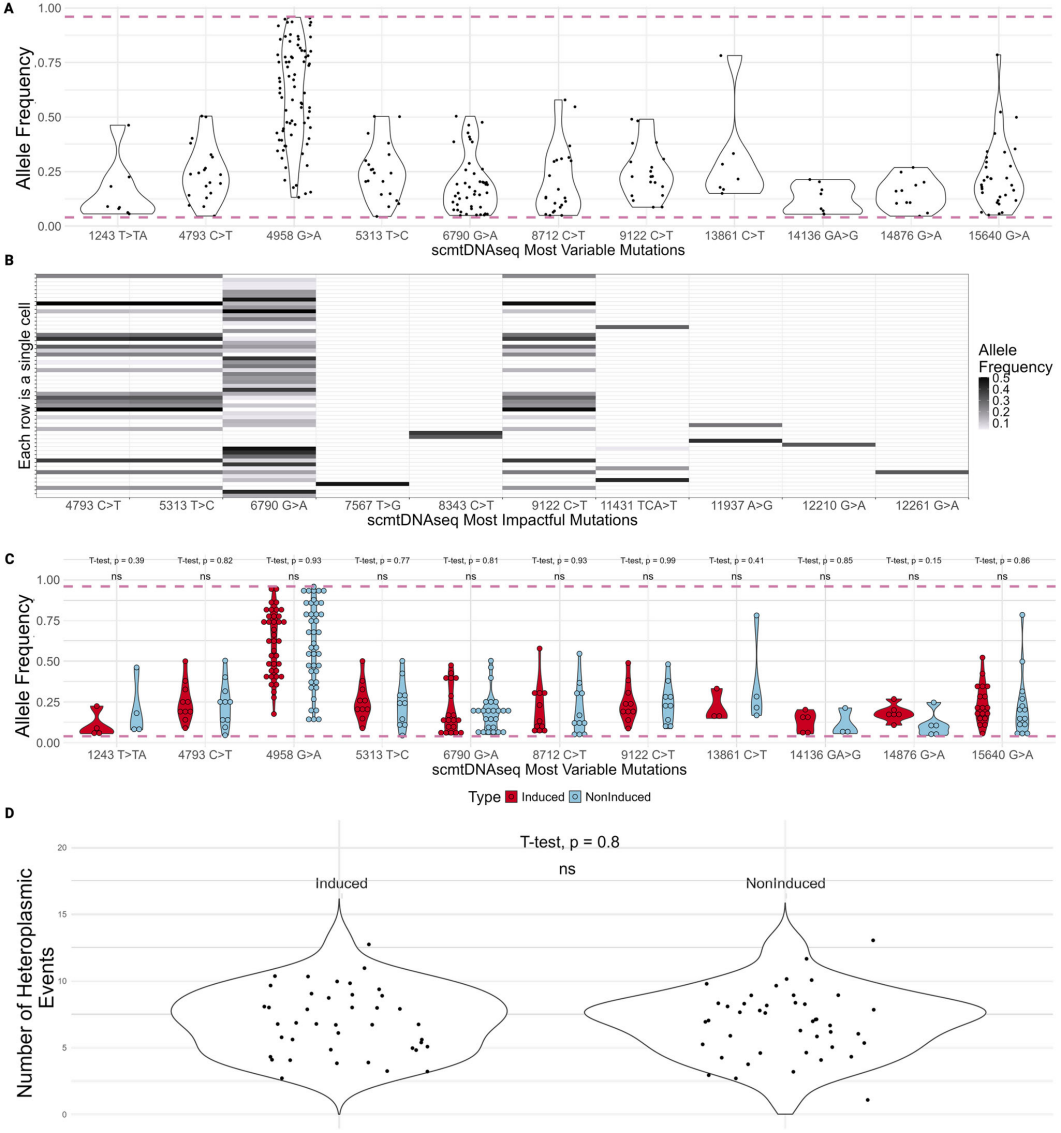
scmtDNAseq analysis. (A) Allele frequency violin plots of “most variable” heteroplasmic mutations observed in scmtDNAseq. Criteria: mutation must be heteroplasmic in at least 5% of cells. (B) Allele frequency heatmap of predicted “most impactful” mutations. Criteria: (1) the mutation is present in at least 1 cell above 30% allele frequency, (2) the mutation is in a coding region and (3) snpEff software algorithm predicts a high or moderate impact; equating to stop‐gained, frameshift, or missense mutations. (C) Allele frequency violin plots of “most variable” heteroplasmic mutations split by induced/noninduced mAb production. (D) Heteroplasmic events per cell violin plot split by induced/noninduced. To investigate heteroplasmic mutations with high levels of variability between cells, we developed a “most variable” mutation list, which had to be heteroplasmic (between 0.04 and 0.96 allele frequency) in at least 5% of cells (Table S1). Eleven mutations were called in the “most variable” list (Figure 3A). We identified that 4958 G>A occurred in 79/84 (94%) of single cells with allele frequency ranging from 0.13 to 0.96. 3/11 (27%) of the “most variable” mutations were not heteroplasmic in bulk samples, reaffirming our previous finding that single cell analysis provides improved resolution [11].

Ten mutations passed these filters (Table S2) (Figure 3B). Six were not identified in bulk samples (Table S1). 57/84 (67.9%) of single cells contained at least one of these mutations. The 6790 G>A mutation was the most common, present in 44/84 (52%) of cells; a stop‐gained mutation in COX1 which would be highly likely to affect OXPHOS and thus cellular processes reliant on abundant ATP generation. Notably, however, all predicted “most impactful” mutations were observed below 0.5 allele frequency, well below what is accepted to be the phenotypic threshold. Interestingly, the mutations 4793 C>T, 5313 T>C, and 9122 C>T always co‐existed and at near identical allele frequencies; apart from one cell where the 9122 C>T mutation dipped slightly below the 0.04 threshold.

### 3.4 Short‐Term Induction of mAb Expression Does Not Affect Heteroplasmy

CHO‐PVZ cells were cultured in flasks in either “induced” or “noninduced” conditions. The inducible nature of the cell line enabled a unique and well‐controlled opportunity to investigate the impact of protein production, in which antibody titer was 9× greater in the induced culture (Figure S4A and S4B). We could therefore directly question whether the burden of mAb production might have an impact on heteroplasmy.

The “most variable” mutations (Table S1) were grouped and compared according to whether they were identified in induced or noninduced culture conditions (Figure 3C). No significant difference was observed in the distribution of heteroplasmy in any of the 11 mutations. We further questioned whether there might be a tendency to accumulate more mutations or for the degree of heteroplasmy at existing loci to change upon induction of high levels of mAb expression. We grouped and compared heteroplasmic events per cell according to whether they were in induced or noninduced mAb‐production conditions (Figure 3D). Once again we observed no significant difference.

For this cell line, we find no evidence of increased mAb production impacting heteroplasmy, albeit over the limited three days the cells were induced to express higher levels of recombinant product. Unfortunately, the induction time could not be extended beyond this due to the cells' tendency to clump, which compromised single‐cell isolation.

### 3.5 scRNAseq Data Can be Used to Identify Heteroplasmy

**FIGURE 4:**
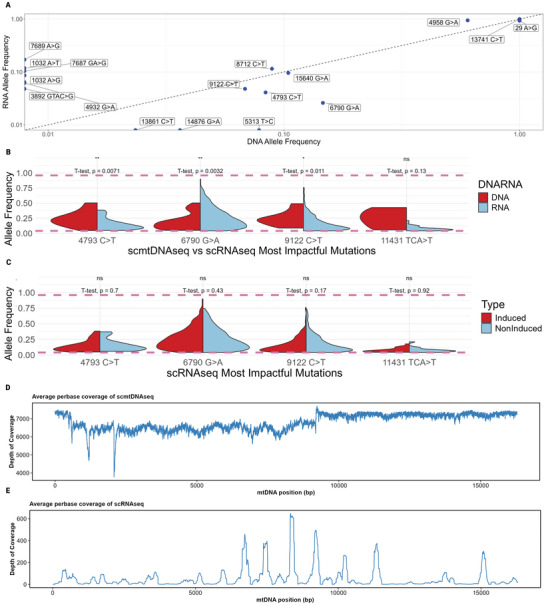
scRNAseq analysis. (A) Annotated allele frequency scatter plot from scmtDNAseq and pseudobulk scRNAseq with an *x* = *y* line in logarithmic scale. (B) Allele frequency violin plot of “DNA and RNA joint most impactful” mutations. Criteria: mutation is in scDNAseq predicted “most impactful” mutation list as in Table S1 and (2) mutation is heteroplasmic in scRNAseq cells. (C) Allele frequency violin plot of only scRNAseq mutations from “DNA and RNA joint most impactful” mutations split by induced/noninduced. (D) Average per‐base coverage of single cells in scmtDNAseq. (E) Average per‐base coverage of single cells in scRNAseq.

scRNAseq provides an opportunity to interrogate much higher cell numbers (over 2000) than scmtDNAseq, though at the cost of lower per base depth (average ∼ 50) (Figure S1F), and only of transcribed regions (Figure 4E). As outlined in Section 2.4, we generated scRNAseq datasets of the PVZ cell line from a different batch of the same experimental setup but without sorting based on AB‐FITC surface staining (Figure 1). To establish whether there were differences in identifying mutations between DNA sequencing and RNA sequencing, we concatenated single cells from our noninduced scRNAseq to produce a pseudo‐bulk sample, which we then compared to a noninduced bulk DNAseq sample at the same time point (Figure 4A). Six mutations were identified to be RNA‐specific. 13861 C>T and 14876 G>A mutations were DNA‐specific and, though present in a small number of scRNAseq cells, the average allele frequency was below 0.04. Mutation 5313 T>C was not present in the scRNAseq data.

We generated a DNA and RNA “joint most impactful” mutation list (Table S1)—whereby the mutation must be (1) in the scmtDNAseq predicted “most impactful” mutation list and (2) heteroplasmic in scRNAseq cells. Only 4/10 (40%) of the mtDNA predicted “most impactful” mutations were heteroplasmic in scRNAseq (Figure 4A,B). Of the “joint most impactful” mutations, there was a significant difference in 3/4 (75%). When splitting the same mutation list by induced/noninduced, no significant difference was observed in any scRNAseq mutations (Figure 4C); however, only mutations with at least 2 cells in each condition were parsed to enable statistical comparison. The number of heteroplasmic events per cell was significantly greater in scRNAseq (Figure S5C). This might reflect the reduced confidence associated with lower per‐base depths. It might also reflect nongenomic events, as later discussed.

A key difference between the scmtDNAseq and scRNAseq methods was the coverage of the mtDNA molecule. Although scmtDNAseq achieved full and deep coverage (Figure 4D), scRNAseq only provided coverage of regions transcribed into RNA and at a lower depth per base (Figure 4E).

### 3.6 Higher Allele Frequencies Were Not Associated with Changes in Gene Expression

The use of scRNAseq provided an opportunity to examine whether higher allele frequencies were associated with changes in gene expression. We focused on the scRNAseq Most Impactful list (Table S1). We first added allele frequency metadata to the scRNAseq dataset, then compared the top quartile of allele frequency with the lowest quartile for each mutation. We then split the datasets into noninduced and induced, to avoid the differentiating factor being induction. We found no differentially expressed genes with *p* adjust < 0.05 for any mutation in either noninduced or induced samples.

We next considered whether there could be a relationship between allele frequency and IgG transcript abundance (Figure S4C). We generated Pearson Correlation tables for each mutation separately for the noninduced and induced datasets. We found no clear correlations (Table S3). We then considered that perhaps a correlation would only be found above phenotypic thresholds. We therefore repeated the analysis only using cells with above 0.6 allele frequency (Table S4). Although some *r* values showed correlation, these were likely highly influenced by the low data number. We therefore concluded there was no clear linear correlation between mutation allele frequencies and IgG transcript abundance.

## 4 Discussion

### 4.1 Minimal Impact of Time on Heteroplasmy

In the biopharmaceutical industry, clonal populations are necessary to ensure “genetic robustness” [20]—and reduce heterogeneity. However, genetic heterogeneity within clonal populations is an inevitability due to DNA‐replication errors; made more pertinent for mtDNA owing to a greater mutation rate when compared to nuclear DNA [21]. Additionally, during mitosis, mitochondria are divided among daughter cells [22]. Uneven distribution over generations can result in different allele frequencies across the population. The many divisions over the course of a standard CHO cell bioprocess run could result in uneven distributions of heteroplasmy by random chance, which may therefore result in heterogeneous phenotypes. Heteroplasmic allele frequencies have previously been observed to dynamically change over a human B‐lymphocyte 28‐day cell culture [23]. In that study, the 15153 G>A allele frequency increased from 0.50 on Day 0, to 0.78 on Day 14 and then decreased to 0.31 on Day 28. In total, 3 mutations revealed a very similar increase‐to‐decrease trend; and their 0.30–0.90 frequency differentials are in a range likely to affect phenotype. Here, negligible changes in heteroplasmy were observed in CHO‐PVZ cells over the duration of a 38‐day culture (Figure 2). As such it would seem that the typical duration of a bioprocess does not significantly impact heteroplasmy. The implication, for the CHO‐PVZ cell line examined here at least, is that the heteroplasmic profile on Day 1 is maintained throughout the 38‐day culture.

However, there was significant intercellular heteroplasmy detected (Figure 3A,B) despite the cell line being clonally derived—i.e., deriving from a single cell progenitor. So, while no temporal effect was detectable, significant variability was observed within the cell population, which clearly must have occurred over time. It would seem that the heteroplasmic differences arose over the time before this experiment—which would have included (1) single cell sorting, (2) clone expansion, (3) freezing, (4) thawing, (5) expansion and routine passaging, and potentially further rounds of (3–5) before initiating this experiment. During this time, mutations could accumulate due to normal exposure to reactive oxygen species (ROS) during respiration as well as during times of stress.

Another explanation is that division of the initial single cell into daughter cells resulted in unbalanced distributions of heteroplasmic mutations to progeny. This is a leading theory as to why heteroplasmic mtDNA mutations can vary hugely from the mother to offspring—a so‐called “genetic bottleneck hypothesis” [24]. This could lead to the development of specific lineages based on their distribution of heteroplasmic mutations. The split from 1 cell to 2 cells would have a much greater effect on variable levels of heteroplasmy within a population since only 2 cells now exist. By contrast, a CHO cell culture doubling from 1 million cells to 2 million cells would not experience the same bottleneck effect. Therefore, we might expect the period after the single cell progenitor to have a much greater impact on differences in intercellular heteroplasmy. So while this study concludes that “38 days has no impact upon intercellular heteroplasmy”, a 38‐day period directly after the initial single cell isolation of the clonal population may have yielded different results.

What is certain is that investigation into cell fate lineages is critical to fully understand the dynamics of heteroplasmy inheritance and by extension their impact on phenotype. Previously, scATACseq was used to investigate clonal tracing of mtDNA [25]. When applied to hematopoietic stem and progenitor cells and peripheral blood mononuclear cells (PBMCs) 257 clonal groups were determined, which correlated with the physiological activity of hematopoietic cells. This suggests that mutations are stably propagated in stem and progenitor cells. The large pool of hematopoietic stem and progenitor cells reduced the impact of individual cells, enabling a “steady state” of hematopoiesis. For CHO cells, can we expect a similar “steady‐state”? Since industrial bioreactors contain billions of cells, the relative impact of a small minority of cells with rare heteroplasmic mutations may be compensated by the vast majority.

### 4.2 Induced mAb Production has a Negligible Impact on Heteroplasmy

The use of an inducible antibody‐producing (palivizumab) CHO cell line enabled a unique, well‐controlled investigation into the impact of recombinant protein production on heteroplasmy. Recombinant protein production has previously been shown to increase a cell's stress response and alter metabolic flux [26]; both likely to involve mitochondria. Furthermore, high‐producing clones were shown to be more frequent during pool selection when mAb production is kept low during nonproduction phases in the cumate‐switch CHO cell line CHOBRI/rcTA [27, 28]. This was attributed to the higher metabolic burden and ER stress associated with high mAb production, resulting in lower survival during pool selection and expansion.

Could this, therefore, extend to affecting heteroplasmy? Here, induced mAb production had a negligible effect on heteroplasmy in both scmtDNAseq and scRNAseq (Figures 3C,D and 4C). Cells were either induced or not on Day 29 and single cells extracted on Day 32. Since cells were only induced for 3 days, it could be that there was not enough time for the increased mAb production to have a measurable impact on heteroplasmy. Future investigations would benefit from longer‐term investigations of induced versus noninduced cell cultures (many passages).

### 4.3 Single Cell mtDNA Analysis Identifies Significant Differences in Intercellular Heteroplasmy

Previous bulk analysis of CHO heteroplasmy obfuscated different single‐cell heteroplasmy conformations [10]. The implications for a CHO cell culture could be important—perhaps only a subset of the cells is working optimally. Perhaps a cell line could be engineered or selected with a favorable heteroplasmy profile. Single‐cell analysis can help address these questions. Previous work demonstrated variability amongst four single cells [11]. Here, the main source of heterogeneity was also revealed to be between individual cells, with the potential to contribute to heterogeneous phenotypes (Figure 3A). 3/11 (27%) “most variable” and 6/10 (60%) predicted “most impactful” heteroplasmic mutations from scmtDNAseq were identified by single cell analysis, and would not have been if only using bulk samples (Table S1). Since phenotypic change is dependent on intracellular allele frequency, we present evidence for novel and potentially impactful mutations that could affect CHO cell performance in the biomanufacturing setting, and more generally intercellular heterogeneity within producing CHO cell lines. This intercellular diversity in heteroplasmy may contribute to the metabolic variability sometimes observed in replicate CHO cell cultures [9]. A further complexity that should be considered is the impact of multiple co‐existing mutations that, individually may be present at low frequencies, but act additively or synergistically with other mutations in the same cell.

### 4.4 scRNAseq as an Alternative to scmtDNAseq

scRNAseq presents a unique opportunity to analyze mtDNA owing to exceptionally high transcriptional coverage and copy number of mitochondrial DNA. The raw sequencing reads, which would usually be used to tally gene counts, can instead be analyzed for sequence variations. Previous investigations of mtDNA using scRNAseq identified heteroplasmic profiles to a sufficient extent to enable cellular lineage tracing [29]. When comparing Whole Genome Sequencing (WGS) to scRNAseq, Ludwig et al. found strong concordance in jointly called mutations. However, RNA‐specific events inferred the presence of nongenomic mutations. There are 4 categories of base variants called from scRNAseq data: (1) genomic mutations, (2) RNA editing events, (3) other RNA modifications, and (4) sequencing errors [29]. Here, widespread nongenomic mutations were observed that were not found by scmtDNAseq. Of the mutations identified by both methods, significant differences were found in allele frequencies (Figure 4A,B). scmtDNAseq, therefore, emerges as preferable to elucidate genuine genomic mutations. Conversely, scRNAseq may also reveal RNA‐editing events, hidden from scmtDNAseq, that could also impact upon phenotype.

One small caveat is that the RNAseq data were not generated from cells sorted for PVZ surface expression, unlike the scmtDNA analysis. Filtering the RNAseq dataset to only cells expressing PVZ transcripts might mitigate this but the relation between mRNA and mature excreted PVZ cannot be assumed.

There is a wider concern in the literature that a mutation identified from scRNAseq may not be a genuine genomic mtDNA mutation. To investigate this, Miller et al. compared their mutation calling from scRNAseq to that using scATACseq libraries [31]. 94.1% of mutations determined by scATACseq above 0.01 allele frequency were also identified in the scRNAseq dataset. Another concern is the impact of Numts from scRNAseq datasets. While the scmtDNAseq method added multiple levels of protection against reads originating from Numts [11], this was not equally achievable in the scRNAseq. Therefore, it is possible that some of the reads originated from the nuclear genome, although previous investigations have suggested mitochondrial RNAs are primarily transcribed from the mitochondrial genome [32].

### 4.5 Conclusions

Potentially, there are technical inaccuracies due to only sequencing a subset of the total mtDNA content in each single cell (sampling error). Additionally, PCR could introduce bias into the mtDNA amplicons, thereby altering allele frequency determinations. However, PCR bias has previously been determined as unlikely to affect the accuracy of allele frequencies in mtDNA analysis [33]. Finally, scRNAseq is useful if large numbers of single cells are to be analyzed but also limited by lack of full mtDNA coverage and, although one might consider transcribed regions of mtDNA as more likely to have phenotypic impacts, noncoding regions can also have an impact [34].

It may be that certain heteroplasmy profiles identified here negatively or positively impact CHO cell bioreactor performance. However, this impact was only predicted in silico and would need experimental validation. To directly link mutations to phenotype, CHO cell lines with specific mtDNA mutations could be compared to wild‐type cultures. Genome engineering using CRISPR/Cas9 has yet to be fully adapted to mtDNA owing to difficulties with RNA transport into mitochondria. However, techniques exist for “heteroplasmy shifting” in which specific mtDNA molecules are targeted using TALENs or Zinc‐finger nucleases resulting in degradation. In successive generations, the remaining mtDNA represents a much greater proportion in cells. By targeting wild‐type mtDNA sequences, it may be possible to generate CHO cell lines with higher allele frequencies of certain beneficial mutations. Alternatively, targeting specific, detrimentally mutated sequences would amplify wild‐type mtDNA. Ultimately, such comparisons might link phenotypic effects to certain mtDNA mutations.

## Author Contributions

**Alan Foley**: conceptualization, data curation, formal analysis, investigation, methodology, project administration, software, visualization, writing – original draft, writing – review and editing. **Nga Lao**: data curation, supervision, writing – original draft. **Ciara McGuirk**: formal analysis. writing – review and editing. **Colin Clarke**: funding acquisition, supervision, writing, review, and editing. **Niall Barron**: conceptualization, funding acquisition, investigation, methodology, project administration, resources, supervision, writing – original draft, writing – review and editing.

## Conflicts of Interest

The authors declare that the research was conducted in the absence of any commercial or financial relationships that could be construed as a potential conflict of interest.

## Supporting information




**Supporting File 1**: biot70079‐sup‐0001‐SuppMat.pdf.

## Data Availability

GitHub repository: https://github.com/alanfoleynibrt/AlanFoleyHeteroplasmyPaper. The bioinformatics pipeline is available in the above GitHub repository. Initial processing of data was performed in Linux, and figures are made in R. All raw FASTQ data and final mutation VCF files are made available. The datasets generated for this study can be found in the SRA accession PRJNA913184.
