# Supplementary material for: Single‐Cell Mitochondrial DNA Analysis of Recombinant Chinese Hamster Ovary Cells Reveals Widespread Heteroplasmy
Source: Biotechnol J. 2025 Sep 1;20(9):e70079. doi: 10.1002/biot.70079 (PMC12400128; doi:10.1002/biot.70079)
Supplement: Supplementary file 1 — Supporting File 1: biot70079‐sup‐0001‐SuppMat.pdf. [file BIOT-20-e70079-s001.pdf]

Supplementary Tables and Figures

Mutation List	Criteria	Mutations
Bulk mtDNA sequencing sample timeline	Must be heteroplasmic in bulk samples along a timeline	[4793 C>T], [4958 G>A], [5313 T>C], [6790 G>A], [8712 C>T], [9122 C>T], [14876 G>A], [15640 G>A]
“most variable”<br><br>scmtDNAseq	Must be heteroplasmic (between 4-96%) and present in at least 5% of cells	[1243 T>TA], [4793 C>T], [4958 G>A], [5313 T>C], [6790 G>A], [8712 C>T], [9122 C>T], [13861 C>T], [14136 GA>G], [14876 G>A], [15640 G>A]
predicted “most impactful”<br><br>scmtDNAseq	Must be present at above 30% in at least 1 cell. Must have snpEff HIGH/MODERATE predicted impact – equating to Frameshift, stop-gained and missense mutations.	[4793 C>T], [5313 T>C], [6790 G>A], [7567 T>G], [8343 C>T], [9122 C>T], [11431 TCA>T], [11937 A>G], [12210 G>A], [12261 G>A]
“joint most impactful”<br><br>scmtDNAseq and scRNAseq	Must be present in scmtDNAseq predicted “most impactful” and in scRNAseq predicted “most impactful”	[4793 C>T], [6790 G>A], [9122 C>T], [11431 TCA>T]

Supplementary Table 1: Mutation lists and their respective criteria

**Mutation**	**Gene**	**Impact (snpEff)**	**Proportion of cells heteroplasmic**	**Proportion of cells above 30% AF**	**Gene function**
4793 C>T<br><br>T**C**T (S) > T**T**T (F)<br><br>Serine > Phenylalanine	ND2	MODERATE: missense	20/84<br><br>23.8%	6/84<br><br>7.1%	Codes for NADH dehydrogenase 2 (NADH-2); part of mitochondrial electron transport and respiratory complex I. Mutations associated with human neurodegenerative diseases myocardial infarction and urinary bladder cancer.
5313 T>C<br><br>**T**TC (F) > **C**TC (L)<br><br>Phenylalanine > Leucine	COX 1	MODERATE: missense	20/84<br><br>23.8%	6/84<br><br>7.1%	Codes for Cytochrome c oxidase 1 (COX-1). Forms part of respiratory complex IV. Involved in electron transport coupled proton transport and electron transport. Mutations associated with human neurodegenerative disease, anaemia, complex IV deficiency, colorectal cancer, sensorineural deafness and myoglobinuria.
6790 G>A<br><br>T**G**A (W) > T**A**A (Stop)<br><br>Tryptophan > Stop	COX 1	HIGH:<br><br>stop gained	44/84<br><br>52.4%	8/84<br><br>9.5%	Codes for Cytochrome c oxidase 1 (COX-1). Forms part of respiratory complex IV. Involved in electron transport coupled proton transport and electron transport. Mutations associated with human neurodegenerative disease, anaemia,

NIBRT Protected Data

					complex IV deficiency, colorectal cancer, sensorineural deafness and myoglobinuria.
7567 T>G<br><br>**T**TT (F) > **G**TT (V)<br><br>Phenylalanine > Valine	COX 2	MODERATE:<br><br>missense	1/84<br><br>1.2%	1/84<br><br>1.2%	Codes for cytochrome c oxidase 2 (COX-2. Part of respiratory chain complex IV. Mutations associated with human Complex IV deficiency.
8343 C>T<br><br>A**C**A (T) > A**T**A (M)<br><br>Threonine > Methionine	ATP 6	MODERATE:<br><br>missense	2/84<br><br>2.4%	2/84<br><br>2.4%	Codes for ATP synthase subunit/chain A. Forms part of the F-type ATP synthase – which performs the last step of OXPHOS in the electron transport chain. Mutations associated with human neurodegenerative diseases and lupus.
9122 C>T<br><br>T**C**A (S) > T**T**A (L)<br><br>Serine > Leucine	COX 3	MODERATE: missense	19/84<br><br>22.6%	5/84<br><br>6.0%	Codes for cytochrome c oxidase III (COX-3); part of respiratory chain complex IV. Mutations associated with human MELAS syndrome.
11431 TCA>T<br><br>**TCA** > **T**CA	ND4	HIGH:<br><br>frameshift	4/84<br><br>4.8%	2/84<br><br>2.4%	Codes for NADH-ubiquinone oxidoreductase chain 4 (ND4); part of NADH dehydrogenase in respiratory chain complex I. Mutations associated with human

NIBRT Protected Data

SNTREL... ><br><br>SHT[Stop]					neurodegenerative disorders, epilepsy, and cystic fibrosis.
11937 A>G<br><br>**<u>A</u>**CA (T) > **<u>G</u>**CA (A)<br><br>Threonine > Alanine	ND5	MODERAT E<br><br>missense	2/84<br><br>2.4%	1/84<br><br>1.2%	Codes for NADH-ubiquinone oxidoreductase chain 5 (ND5); part of NADH dehydrogenase in respiratory chain complex I. Mutations associated with human neurodegenerative disorders, encephalomyopathy, MELAS and Leigh’s syndrome.
12210 G>A<br><br>**<u>G</u>**CA (A) > **<u>A</u>**CA (T)<br><br>Alanine > Threonine	ND5	MODERAT E<br><br>missense	1/84<br><br>1.2%	1/84<br><br>1.2%	Codes for NADH-ubiquinone oxidoreductase chain 5 (ND5); part of NADH dehydrogenase in respiratory chain complex I. Mutations associated with human neurodegenerative disorders, encephalomyopathy, MELAS and Leigh’s syndrome.
12261 G>A<br><br>**<u>G</u>**GC (G) > **<u>A</u>**GC (S)<br><br>Glycine > Serine	ND5	MODERAT E<br><br>missense	1/84<br><br>1.2%	1/84<br><br>1.2%	Codes for NADH-ubiquinone oxidoreductase chain 5 (ND5); part of NADH dehydrogenase in respiratory chain complex I. Mutations associated with human neurodegenerative disorders, encephalomyopathy, MELAS and Leigh’s syndrome.

NIBRT Protected Data

Supplementary Table 2: List of predicted "most impactful" mutations from scmtDNAseq with position in KX576660.1 CHO mtDNA reference genome, base and amino change, gene, snpEff predicted impact, allele frequency, gene function and associated mutations (Alliance of Genome Resources, 2023) (OMIM, 2023).

mutation	pvz_choice	cor_HC_r	cor_HC_p	cor_LC_r	cor_LC_p
4793_AF	pvz_n	-0.07	0.544	-0.02	0.842
6790_AF	pvz_n	0	0.973	-0.05	0.448
9122_AF	pvz_n	0.03	0.743	-0.07	0.416
11431_AF	pvz_n	-0.13	0.682	-0.4	0.192
4793_AF	pvz_i	0.13	0.209	-0.04	0.688
6790_AF	pvz_i	0.06	0.339	-0.04	0.562
9122_AF	pvz_i	0.01	0.914	-0.02	0.81
11431_AF	pvz_i	-0.08	0.762	0.17	0.532

Supplementary Table 3: Pearson Correlations of mutation allele frequencies with heavy and light chain IgG expression levels. R value: 1 = perfect positive linear correlation, -1 = perfect negative linear correlation and 0 = no correlation. P values included.

NIBRT Protected Data

mutation	pvz_choice	cor_HC_r	cor_HC_p	cor_LC_r	cor_LC_p
4793_AF	pvz_n	-0.54	0.457	0.84	0.161
6790_AF	pvz_n	0.1	0.706	-0.19	0.473
9122_AF	pvz_n	0.03	0.967	-0.68	0.324
11431_AF	pvz_n				
4793_AF	pvz_i				
6790_AF	pvz_i	0.54	0.108	0.09	0.814
9122_AF	pvz_i				
11431_AF	pvz_i				

Supplementary Table 4: Pearson Correlations of mutation allele frequencies with heavy and light chain IgG expression levels but only for allele's above 0.6 frequency. Missing values indicate too few data points for Pearson Correlation.

NIBRT Protected Data

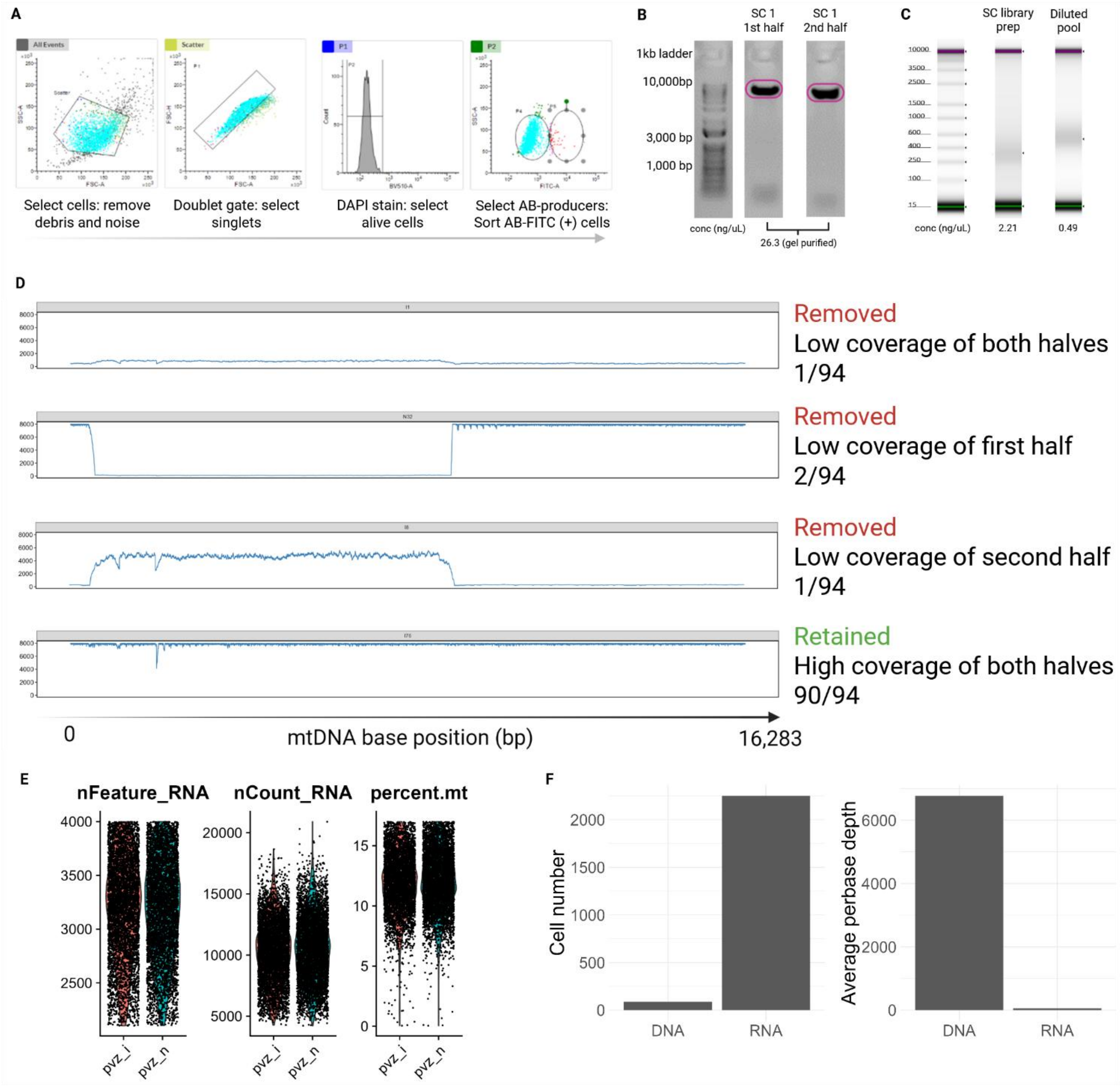

Supplementary Fig. 1: (A) FACS Melody graphs to sort single cells for scDNAseq. **(B)** Gel electrophoresis to display successful amplification of DNA from a single cell in 2 halves. **(C)** TapeStation trace confirming successful Illumina DNA Prep. **(D)** Quality control of scDNAseq based on perbase coverage (84 single cells taken forward, 4 removed, 6 bulk samples, 84 + 4 + 6 = 94). **(E)** Quality control of scRNAseq to select healthy live cells and singlets. Cutoffs: mitochondrial reads<17% and 2100<nFeature_RNA<4000. **(F)** Differences in cell number and average perbase coverage between scDNAseq and scRNAseq.

NIBRT Protected Data

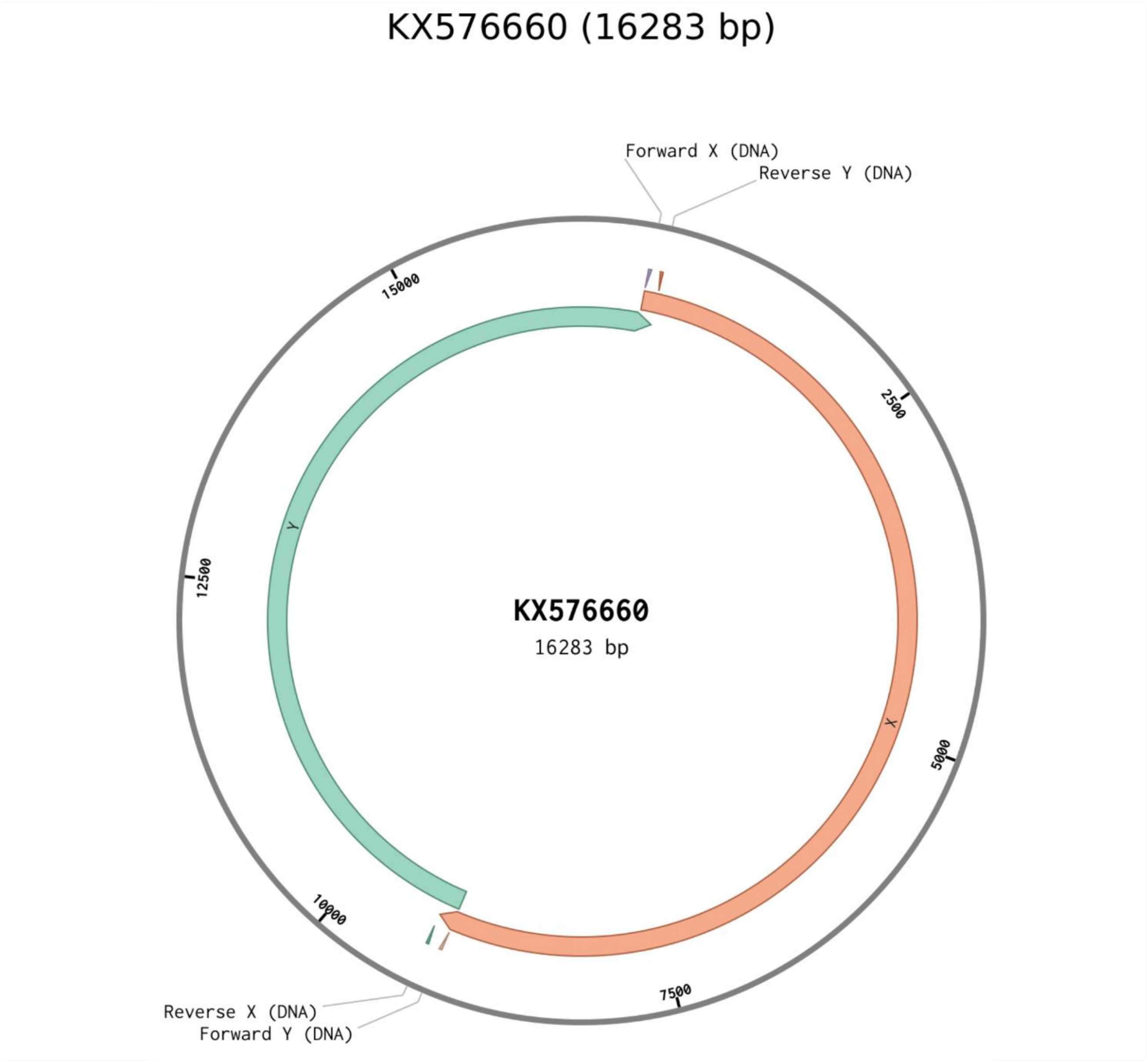

Supplementary Fig. 2: The KX576660 CHO mtDNA molecule with annotated long-range PCR fragments X and Y as previously performed (Foley et al., 2024)

NIBRT Protected Data

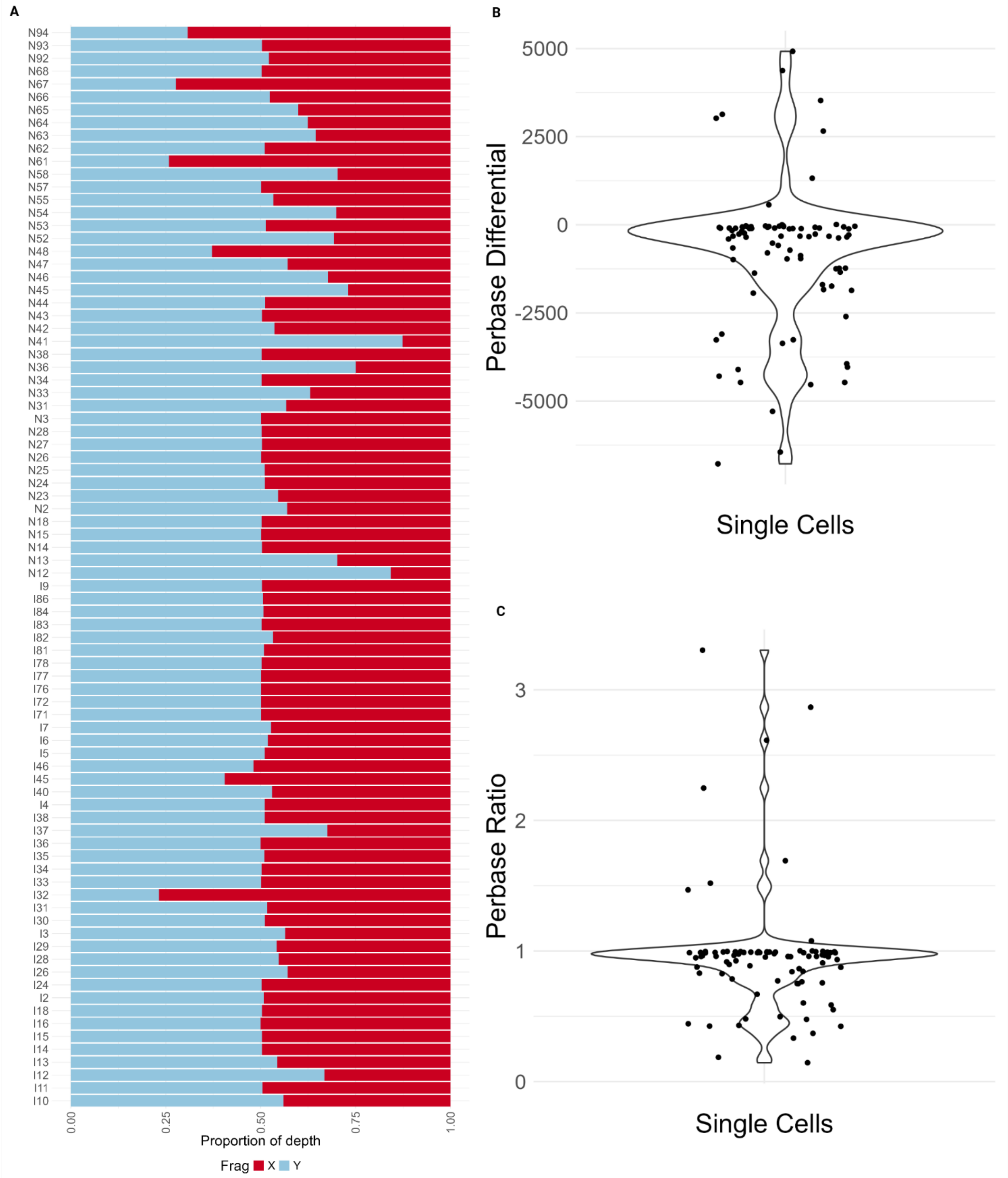

Supplementary Fig. 3: Identifying differences in read depth from the separate fragments from scDNAseq. **(A)** The proportion of depth for each fragment. **(B)** The differential between X and Y (X – Y). **(C)** The ratio of depths between X and Y (X / Y).

NIBRT Protected Data

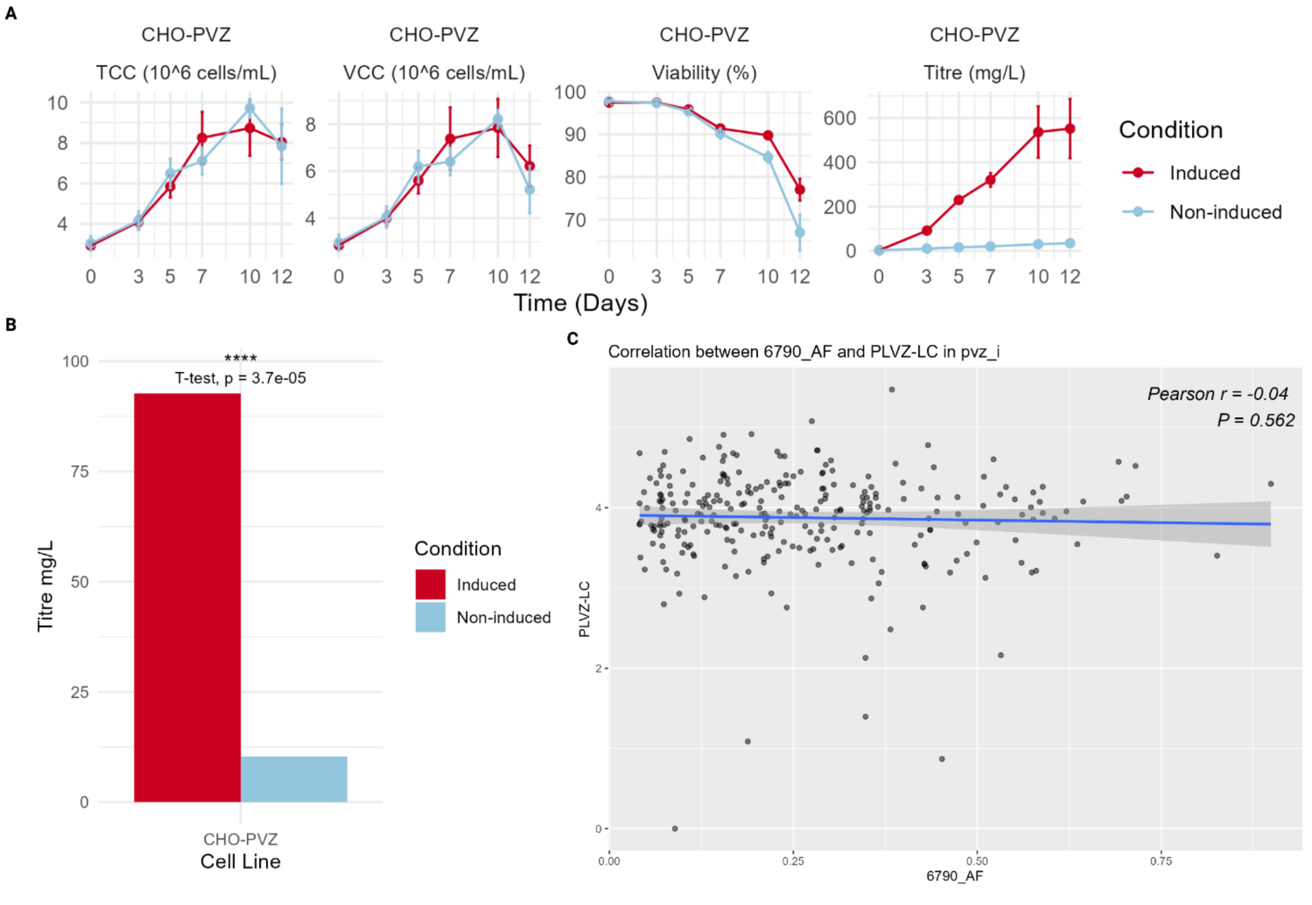

Supplementary Fig. 4: (A) Total Cell Concentration (TCC), Viable Cell Concentration (VCC), Viability and Titre of the CHO-PVZ cell line after induction. Mean values with standard deviation. **(B)** Titre on Day 3. (**C**) An example of allele frequency correlation analysis with LC gene expression from scRNAseq (PVZI only).00

NIBRT Protected Data

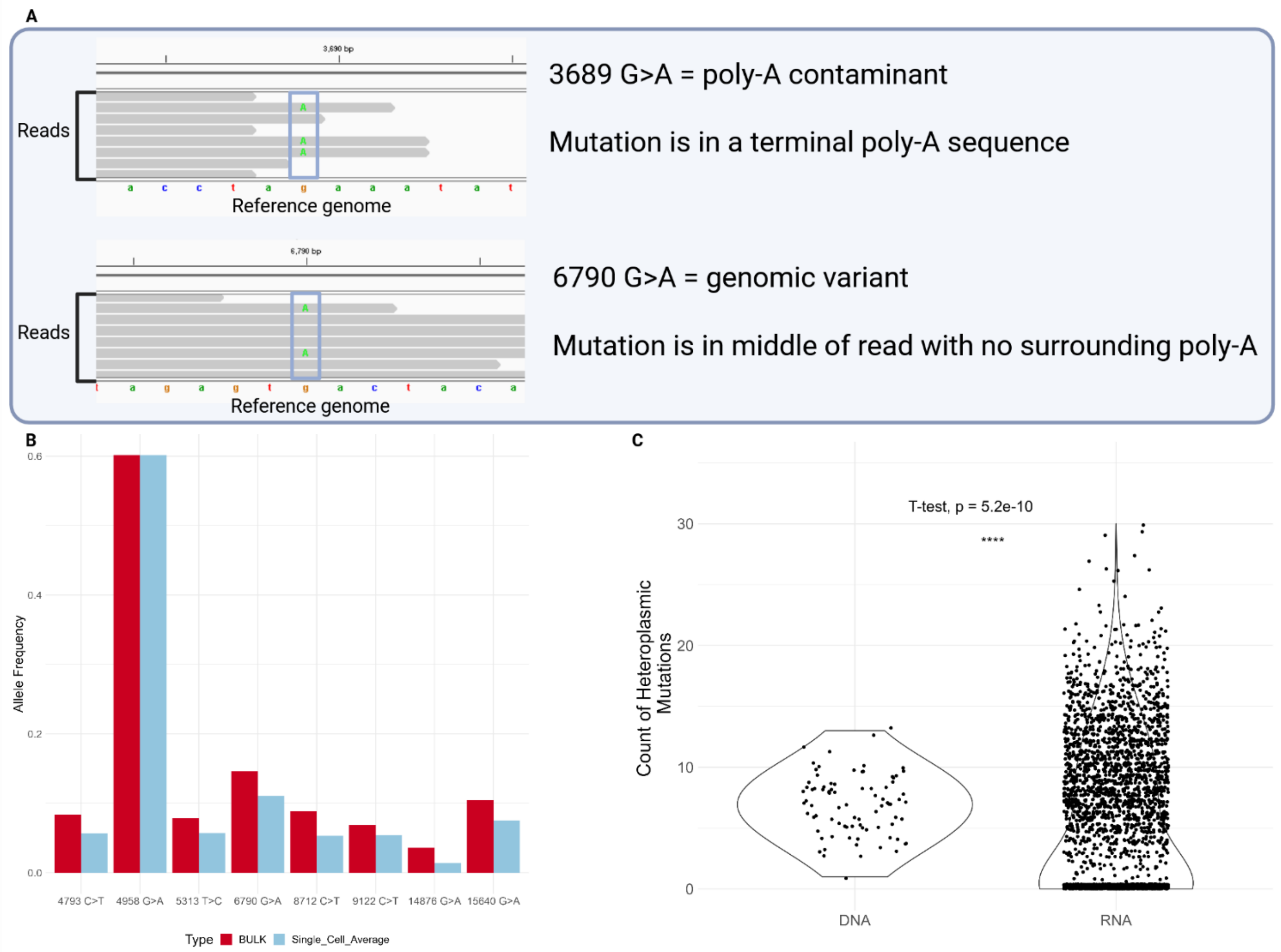

Supplementary Fig. 5: **(A)** Discriminating genuine and poly-A contaminating N>A mutations from scRNAseq mutation calling. **(B)** Allele frequency bar chart comparing averaged scDNAseq single cells to a bulk DNAseq sample. **(C)** Violin plot to compare the count of heteroplasmic mutations from scmtDNAseq and scRNAseq.
